# Tuning Superhydrophobic Materials with Negative Surface Energy Domains

**DOI:** 10.34133/2019/1391804

**Published:** 2019-11-30

**Authors:** Zhongzhen Wu, Liangliang Liu, Shunning Li, Shunping Ji, Pinghu Chen, Suihan Cui, Zhengyong Ma, Yuchang Weng, Qian Huang, Zhongcan Wu, Hao Wu, Yuan Lin, Ricky K. Y. Fu, Hai Lin, Xiubo Tian, Paul K. Chu, Feng Pan

**Affiliations:** ^1^School of Advanced Materials, Peking University Shenzhen Graduate School, Shenzhen 518055, China; ^2^Department of Physics and Materials Science, City University of Hong Kong, Tat Chee Avenue, Kowloon, Hong Kong, China

## Abstract

Hydrophobic/superhydrophobic materials with intrinsic water repellence are highly desirable in engineering fields including anti-icing in aerocrafts, antidrag and anticorrosion in ships, and antifog and self-cleaning in optical lenses, screen, mirrors, and windows. However, superhydrophobic material should have small surface energy (SE) and a micro/nanosurface structure which can reduce solid-liquid contact significantly. The low SE is generally found in organic materials with inferior mechanical properties that is undesirable in engineering. Intriguingly, previous theoretical calculations have predicted a negative SE for *θ*-alumina (*θ*-Al_2_O_3_), which inspires us to use it as a superhydrophobic material. Here, we report the experimental evidence of the small/negative SE of *θ*-Al_2_O_3_ and a *θ*-Al_2_O_3_-based superhydrophobic coating prepared by one-step scalable plasma arcing oxidation. The superhydrophobic coating has complete ceramic and desired micro/nanostructure and therefore exhibits excellent aging resistance, wear resistance, corrosion resistance, high-temperature tolerance, and burning resistance. Owing to the rarity of the small/negative SE in inorganic materials, the concept to reduce SE by *θ*-Al_2_O_3_ may foster a blowout to develop robust superhydrophobicity by complete inorganic materials.

## 1. Introduction

From the view of thermodynamics, the SE of solid materials in a single-component cannot be negative; otherwise, they will sublimate. However, in a multicomponent system, the SE depends strongly on the chemical environment as follows:

(1) $$\begin{array}{c} \gamma =\frac{1}{A}\left(E_{\text{surf}}-\sum N_{i}\mu_{i}\right), \end{array}$$

where *E*_surf_ is the total energy of the system for the surfaces; *A* is the surface area; *N*_*i*_ and *μ*_*i*_ are the number and the chemical potential of constituent element *i*. When no external element exists, the surface energy will correspond to the free energy gain in creating the dangling bonds. If external elements are incorporated into the system, surface reconstruction occurs by for example passivation, adsorption, or hydration, and the newly formed chemical bonds will compensate the energy of the dangling bonds, thus lowering the surface energy to a value that possibly goes below zero after deducting the heat of the adsorbate.

**Figure 1:**
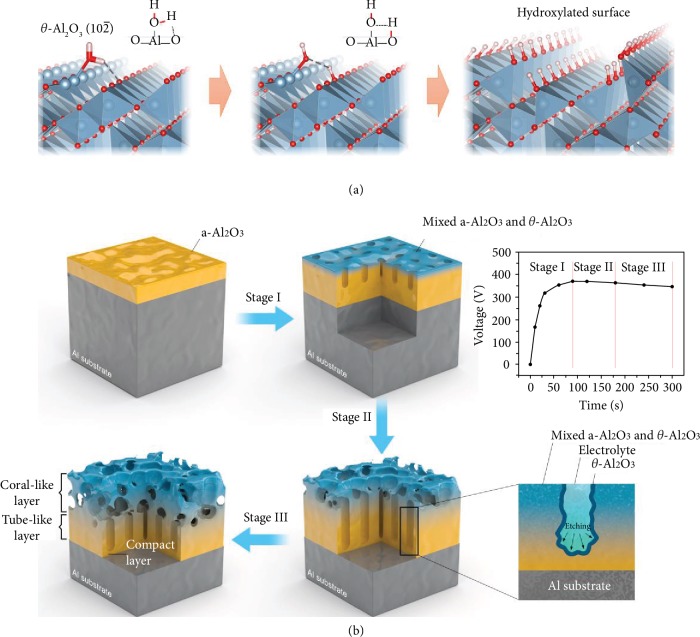
(a) Schematic illustration of the hydration process. Water molecules will dissociate into OH- and H- fragments, releasing energy that can compensate the energy penalty caused by the cleavage of Al-O bonds. The overall surface energy can become negative owing to this compensation effect. (b) Electrochemical mechanism of the coating growth. Three stages are shown: (I) Al dissolution and alumina formation, (II) Al_2_O_3_ erosion, and (III) formation of the coral-like layer.

Typically, in 2004, Lodziana et al. discovered that the surfaces of *θ*-Al_2_O_3_ have much lower SE after hydration, especially the (10-2) plane which has a negative SE by DFT calculation [1]. The negative surface energy of *θ*-Al_2_O_3_ is induced by the dissociation of water molecules over the (10-2) surfaces, as shown in Figure 1(a). When a water molecule is placed on stoichiometric *θ*-Al_2_O_3_ (10-2) surface, with its O atom at a distance allowing the formation of chemical bonds to one of the exposed Al atoms, a dissociation reaction into OH- and H- fragments would be energetically favored. The OH- fragment remains on top of the Al atom while the H- fragment is transferred onto the surface of a nearby O atom. Since no excess charge is introduced in this process, all the Al and O atoms in the surface layer of *θ*-Al_2_O_3_ could be fully covered by OH- and H-, respectively. By this, the difference in chemical environment between the surface and the bulk atoms would be largely eliminated, relieving the internal stress built up during the creation of these surfaces. The saturation of dangling bonds with hydroxyl species will compensate the energy consumption for the cleavage of Al-O bonds, leading to substantially low or even negative surface energy. The high stability of the hydroxylated surfaces would eventually shape the preference for a high-surface-area micro/nanostructures as the thermodynamic ground state, which guarantees their resistance to structural collapse.

The small or negative SE of the hydroxylated *θ*-Al_2_O_3_ [1] suggests a new modification layer to obtain superhydrophobicity, which is inorganic and has good mechanical properties. However, *θ*-Al_2_O_3_ is a metastable polymorph obtained in the heating process to synthesize *α*-Al_2_O_3_ and it cannot preserve to room temperature unless it exists as nanograins and coexists with other alumina phase. Although previous work reveals the hydrophobicity of Al_2_O_3_ by plasma electrolytic oxidation (PEO) [2], it is far from the regular structure and *θ*-Al_2_O_3_. Here, we employ the arcing discharge plasma chemistry in PEO and surface structure control in anodic oxidation [3] to develop a one-step and scalable method to prepare a multiphase (*θ*, amorphous, etc.) intergrowth composite Al_2_O_3_ coating with the coral-like micro/nanostructure which has a surface layer composed of nanosize *θ*-Al_2_O_3_ grains and micro/nanopores. The observation of superhydrophobicity is reported on this coating surface, which offers excellent performance under harsh conditions including high temperature, open flame, corrosion, wearing, and aging.

A high temperature of about 2,000°C produced by instantaneous arcing in PEO can provide enough energy to form *θ*-Al_2_O_3_, while the shock cooling in the water-based electrolyte cools *θ*-Al_2_O_3_ to room temperature and creates conditions for hydration [4]. Inspired by the controllable micro/nanostructures by pH in anodic oxidation [5], a weakly acidic electrolyte is adopted to produce the nanoporous structure. At the same time, NaF is introduced to the electrolyte to produce HF slowly as the microarc discharge energy is increased in the plasma discharge. HF etches the surface of the multiphase alumina coating [6] producing a thin surface layer with mainly *θ*-Al_2_O_3_.

## 2. Method

### 2.1. Sample Preparation

The commercial LY12 Al alloy was cut into rectangular pieces with dimensions of 50 mm × 25 mm × 1 mm. The samples were degreased in acetone and dried in warm air. A 20 kW pulsed bipolar power supply and constant current mode were used in the PEO experiment, and the electrolyte was composed of sodium hexametaphosphate ((NaPO_3_)_6_, 20 g/L), ethylenediamine tetraacetic acid disodium salt (Na_2_EDTA, 5 g/L), sodium fluoride (NaF, 3 g/L), and ammonium iron(III) oxalate hydrate (C_6_H_12_FeN_3_O_12_·3H_2_O, 4.5 g/L). The experiments were performed at a constant current density (6 A dm^−2^) at 200 Hz for 5 min with a duty cycle of 20% using a 20 kW AC power supply. The temperature of the electrolyte was controlled to be below 40°C using an external water cooling system. After PEO, the samples were washed with distilled water and vacuum-dried at 120°C for 24 hours before the contact angle measurements.

### 2.2. Sample Characterization

A field emission scanning electron microscope (FESEM, Carl Zeiss, SUPRA® 55) was employed to characterize the surface and cross-sectional morphologies of the PEO coatings and the elemental distribution and composition were determined by energy-dispersive X-ray spectroscopy (EDS). The phase composition was determined by X-ray diffraction (XRD, Bruker, D8 Advance) with a Cu target (*λ* = 0.15418 nm) and the data were collected at 2*θ* = 10°~80° at a scanning rate of 2*θ* = 2°/min. X-ray photoelectron spectroscopy (XPS, Thermo Fisher, ESCALAB 250X) was utilized to determine the composition of the coatings, and the spectra were referenced to the C1s peak (284.8 eV). The atomic structure of the coating was examined by high-resolution field emission transmission electron microscope (TEM: JEM-3200FS, Japan).

### 2.3. Performance Measurements

#### 2.3.1. Contact Angle Measurements

The water CAs were measured on a commercial contact angle meter (PT-705B, Dong Guan Precise Test Equipment Co., Ltd., China). The volume of water droplets was 2 *μ*L, and three different spots were measured on each sample to obtain the average.

#### 2.3.2. Aging Testing

The samples were exposed to air for 360 days, and the contact angle was measured every 30 days. The CAs shown here are the average of 5 measurements.

#### 2.3.3. Wear Testing

The friction and wear tests were conducted on a reciprocating machine (MFT-5000, Rtec Instruments, USA) with the nonwoven fabric as the counterpart. The load was 10 N, friction frequency was 0.5 Hz, and wear distance was 15 mm. The CAs and surface morphology were determined after 2,000 cycles at ambient temperature (relative humidity is about 30%).

#### 2.3.4. Corrosion Testing

The polarization curves were acquired on an electrochemical workstation (1470E, Solartron Metrology) between -2.0 and 0 V versus the saturated calomel electrode (SCE) at a scanning rate of 10 mV/s at 25°C in the NaCl (3.5 wt%) solutions. The corrosion potential (Ecorr) and corrosion current density (icorr) were analyzed by the Tafel extrapolation method. The salt spraying test was performed to evaluate the CAs on the coatings and dependence on corrosion time.

#### 2.3.5. Firing Testing

Three types of materials including polyimide (PI), polytetrafluoroethylene (PTFE), and the superhydrophobic coating were examined in an alcohol lamp flame to evaluate the flame resistance and the hydrophobicity was evaluated.

## 3. Results and Discussion

The U-I curves of the discharge and schematic of the coating mechanism are shown in Figure 1(b). In the constant current mode, the oxide layer thickens with discharge time and the impedance increases. As a result, the discharge voltage should increase monotonically until the end of the discharge. However, in the weakly acidic electrolyte, the discharge voltage increases quickly at first but decreases slowly when the voltage is over 400 V, revealing three stages in the coating process. At the beginning of PEO, alumina grows on the Al alloy initially and a larger discharge intensity and arcing temperature increase the coating thickness. Transmission electron microscopy (TEM) suggests that the multiphase alumina including the *θ* phase begins to form ([Supplementary-material supplementary-material-1]) [4]. As the coating becomes thicker and the impedance increases, the total arc number decreases, while the intensity of each arc increases [5]. The higher arc energy improves the corrosion of the coating in the weakly acidic electrolyte resulting in decreased discharge voltage. Therefore, both growth and corrosion (etching) take place and the nanotubular structure starts to from in the coating in the second stage. Although the impedance of the coating decreases slightly because of the porous structure, the thickness of the coating still increases with time. The arc energy continues to increase inducing the reaction between NaF and the acidic electrolyte and subsequent production of HF. Owing to the reduced arc number, the amount of HF is small and so only amorphous alumina, not crystalline, is etched [7]. In this way, crystalline alumina (mainly *θ*-Al_2_O_3_) domain which has high corrosion resistance is formed in the coating surface. The pH and the NaF concentrations are optimized and the CAs are shown in [Supplementary-material supplementary-material-1].

**Figure 2:**
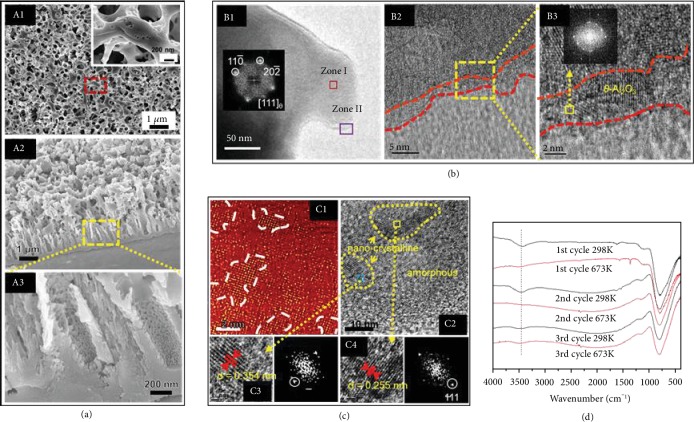
Morphology and structure of the prepared coating. (a) Surface and the cross-sectional images by SEM showing three typical layers form the surface to substrate: irregular coralline-like layer, nanotubular layer, and dense layer. (b) TEM images of the powder scraped from the coating: a 2-5 nm crystalline layer (mainly *θ*-Al_2_O_3_) is observed on the surface of the coating. (c) High-resolution images of the *θ*-Al_2_O_3_ grains and electron diffraction data: *θ*-Al_2_O_3_ (111) and (10-2) nanograins are embedded in the amorphous alumina matrix. (d) IR spectrum at room temperature and 673 K: the hydration/dehydration repetition shows a repeatable small SE and superhydrophobicity.

Figure 2 and [Supplementary-material supplementary-material-1] show the materials and structure of the coatings. The coating surface exhibits an irregular coralline-like interconnected skeleton forming a cellular structure with a size of 300 to 400 nm. In each skeleton, small nanoholes with a size of 10-50 nm are present. This multilevel micro/nanostructure ([Supplementary-material supplementary-material-1]) shows the corrosion effects of the weakly acidic electrolyte. The cross-sectional images show three typical layers between the surface and substrate with a total thickness of 4-5 *μ*m. The top layer has an irregular coralline-like structure, and the middle layer has a nanotubular structure with a diameter of about 200 nm and 10-50 nm nanoholes. The bottom layer is a dense layer with a thickness of about 300 nm ([Supplementary-material supplementary-material-1]). The coating is mainly composed of Al and O and a trace amount of C which arises from surface adsorption of hydrocarbon compounds from air. No obvious peaks are observed by XRD, thus suggesting an essentially amorphous structure ([Supplementary-material supplementary-material-1]). The results show that an alumina coating with a multilevel micro/nanostructure is formed. TEM performed on the coating materials scraped off from the sample shows that the coating contains not only amorphous but also crystalline alumina distributed uniformly in the amorphous alumina matrix as nanograins with a size of 2-5 nm. The d-spacings of the alumina grains determined by electron diffraction (Figure 2(c)) are 0.255 nm and 0.354 nm [8], corresponding to the *θ*-Al_2_O_3_ (111) and [10-2], respectively, which are the planes with small or negative SE after hydration [1, 9]. A 2-5 nm crystalline layer is observed from the surface of the alumina particles as shown in Figure 2(b). It is composed of *θ*-Al_2_O_3_ and expected to play a critical role in the surface hydrophobicity. The coating materials are scraped from the substrate and pressed them into a bulk disc for testing ([Supplementary-material supplementary-material-1]). The disk shows a contact angle of about 120° confirming a low SE surface.

The surface hydrophobicity of the coatings depends on hydration/dehydration of *θ*-Al_2_O_3_ which plays an important role to produce smaller or negative SE. The dehydration temperature is about 623 K as measured by infrared spectroscopy (IR) spectrum in [Supplementary-material supplementary-material-1]. The hydrophobicity of the hydration/dehydration coating is performed, as shown in Figure 2(d). At the room temperature, the IR spectrum taken from the powder scraped from the coating shows an obvious vibration peak at 3489 cm^−1^ which is the typical vibration of OH-, suggesting that the *θ*-Al_2_O_3_ is hydroxylated. When the coating is heated to 673 K, IR shows that the vibration peak of OH- disappears indicating dehydration. At the same time, the coating becomes hydrophilic due to increased surface energy from dehydration [10]. However, the dehydrated *θ*-Al_2_O_3_ can regain superhydrophobicity after hydration again. The IR spectrum of the dehydrated coating after hydration for 24 h in air shows the OH- peak again implying recovery of the hydrophobicity. This phenomenon repeats itself with heating thereby providing evidence that hydration plays a critical role in the small or negative SE and superhydrophobicity of *θ*-Al_2_O_3_.

**Figure 3:**
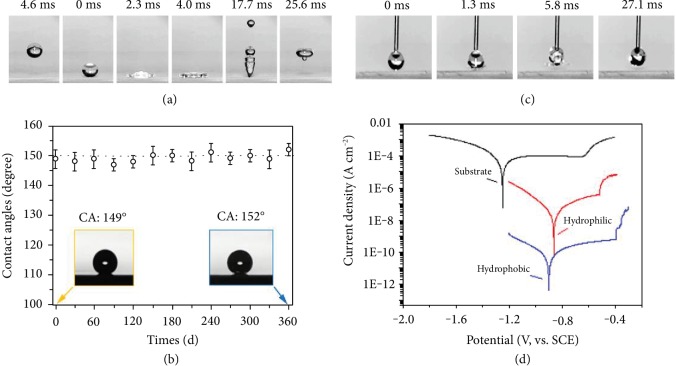
(a) Droplet bouncing test: the droplet cannot spread but instead bounces as it contacts the coating surface, (b) aging test showing the long-time performance of the coating, (c) high-temperature test at 300°C, and (d) corrosion test showing the excellent corrosion resistance.

The wetting experiment on the coating shows that the static contact angle is 152° and the sliding angle is no more than 3°. The droplet cannot spread but instead bounces as it contacts the coating surface as observed by high-speed photography (Figure 3(a), [Supplementary-material supplementary-material-1], and [Supplementary-material supplementary-material-1]). In comparison, a hydrophilic surface wets readily and the droplet does not bounce. To assess the robustness of superhydrophobicity, aging, wear, high-temperature, and burning experiments are performed. After ambient exposure for one year, no obvious decrease in the contact angle occurs as shown in Figure 3(b). The high-temperature experiment is conducted by adding a heater to the contact angle instrument ([Supplementary-material supplementary-material-1] and [Supplementary-material supplementary-material-1]). The sample is placed on the heating stage and heated to 300°C. At this high temperature, the droplet spreads quickly upon contact with the hydrophilic material surface. A cone forms from the needle to the sample surface and a large amount of bubbles are produced until the droplet evaporates. However, when the droplet makes initial contact with the prepared coating surface as shown in Figure 3(c), it bounces immediately and does not stay on the surface suggesting the occurrence Leidenfrost Phenomenon which means that an insulating vapor layer on the coating surface is produced and keeps the liquid from boiling rapidly [11]. The different phenomenon between the hydrophilic surface and prepared coating reveals that the superhydrophobicity decreases the temperature threshold of Leidenfrost Phenomenon to <300°C. Compared to the organic materials, these types of inorganic materials have significant advantages concerning fire resistance. In order to check if F contained organics during synthesis retain on the coating surface, the burning experiments are performed on the coating and compared with polyimide (PI) and polytetrafluoroethylene (PTFE), as shown in [Supplementary-material supplementary-material-1] and [Supplementary-material supplementary-material-1]. The PI sample ignites after 3 s on the outerflame of the alcohol lamp. Although PTFE is an important refractory material, it does not ignite but melts completely after 12 s. In comparison, the coating keeps the excellent superhydrophobicity after 18 s firing. Besides, the burning test makes sure further that no F contained organics retain on the coating surface because of their small ignition temperature.

The coating has good wear resistance, and the contact angle remains over 130° after 2,000 cycles of the friction test using nonwoven fabric at a load of 10 N, as shown in [Supplementary-material supplementary-material-1]. The good wear resistance is mainly attributed to the mechanical performance of alumina but the loose and nanoscale coral-like structure can collapse under a erally have good corrosion resistance because of water repellence. The corrosion resistance of the coating is assessed electrochemically and by salt spraying. Generally, it is difficult to obtain electrochemical data because an air film forms between the coating and corrosive solution [12]. In order to destroy the air film, the coating is immersed into a corrosive solution for 24 h before the electrochemical test as shown in Figure 3(d). The corrosion current decreases significantly and is 4 and 2 orders of magnitude smaller than those of the substrate and hydrophilic coatings, respectively, thereby showing excellent corrosion resistance. Salt spraying reveals the similar corrosion resistance, and the contact angle is still above 140° after 250 h and 370 h as shown in [Supplementary-material supplementary-material-1]. It is noted that the contact angle decreases mainly during initial corrosion possibly due to destruction of the air film and is nearly constant afterwards.

## 4. Conclusions

**Figure 4:**
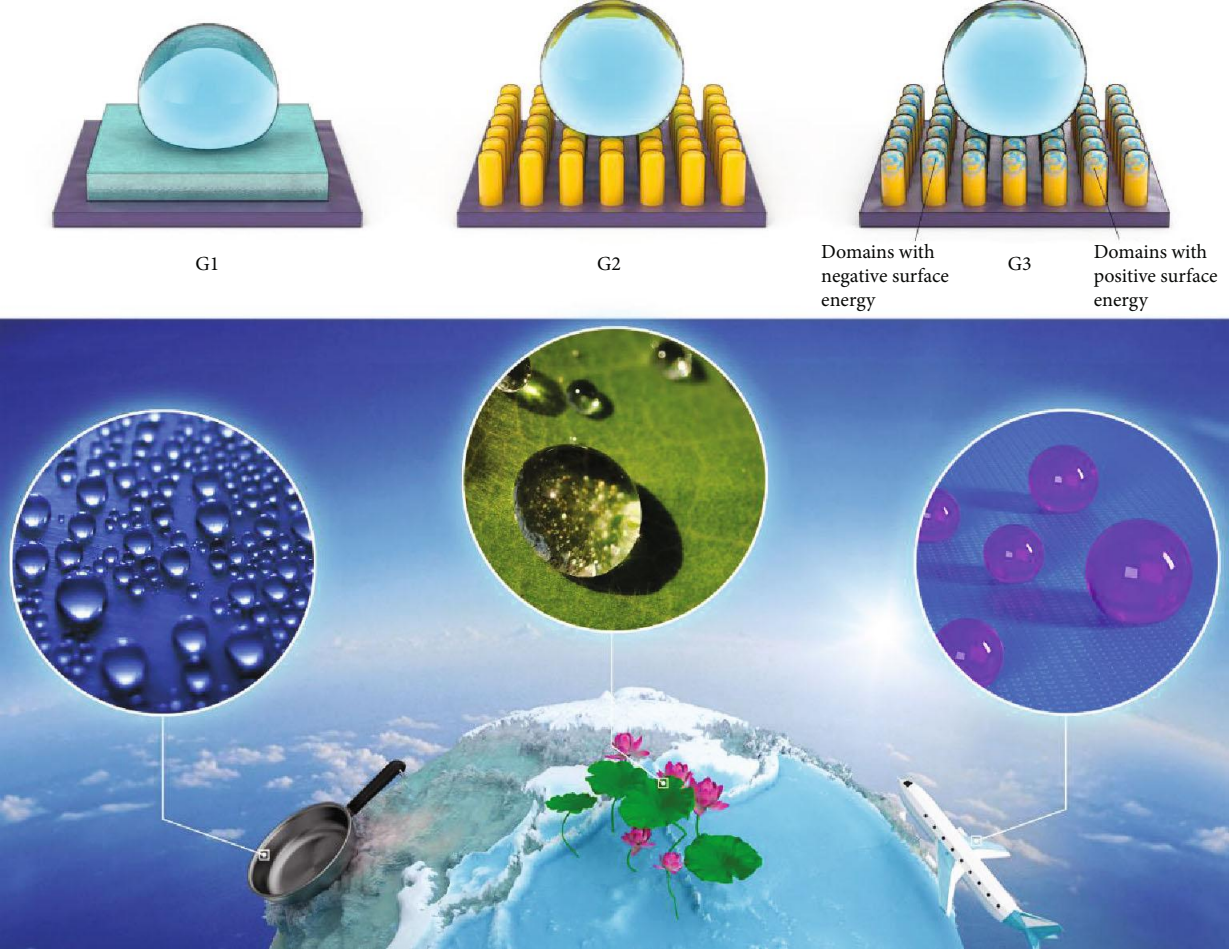
Schematic showing the three types of hydrophobic materials: G1: Si and F containing organic materials have a smooth surface and low surface energy and the contact angle is between 90° and 119°; G2: periodic micro/nanostructures with alternate Si, and F containing organic materials and air form a super hydrophobic surface and the contact angle is above 150°; G3: periodic micro/nanostructure containing alternating air and inorganic materials with the average surface energy reduced by negative surface energy materials provide superhydrophobicity along with high mechanical strength.

Surface adsorption and hydroxylated surface states can decrease the surface energy of inorganic materials, as demonstrated by previous studies on rare-earth oxides [13, 14]. On the smooth surface, the contact angle of water droplets can be larger than 90° exhibiting hydrophobicity [10]. According to Young's theory, the contact angle has a theoretical limit and no larger than 119°on a smooth surface morphology (lowest SE = 6.7 mJ/m^2^). Therefore, the low SE materials (generally organic compounds containing F and Si) with contact angles between 90° and 119° on a smooth surface morphology can be categorized as first-generation hydrophobic materials as shown in Figure 4. To develop superhydrophobic materials (CA > 150°) with self-cleaning effects, periodic micro/nanostructures inspired by natural materials such as lotus leaves have been proposed [15–17]. This kind of superhydrophobic surface with the alternating composition of low SE materials and air, which can be considered to have zero SE, is classified as second-generation hydrophobic materials (G2). However, organic materials tend to have inferior mechanical properties and aging resistance, thus plaguing wider engineering applications [18, 19]. Only a few kinds of inorganic materials, such as ZnO and TiO_2_, are reported to have superhydrophobicity after interface reconstruction by hydroxyl group adsorption. Until now, no synthetic surface reported can possess both high mechanical strength and superhydrophobicity, except the multiphase Al_2_O_3_ composed of *θ*-Al_2_O_3_ fabricated in this work. Tuning the multiphase Al_2_O_3_, which decrease the SE of a strong material by another strong material with small or negative SE, together with the alternating bulk materials and air, prompts us to term this the third-generation hydrophobic material (G3). It is anticipated that this type of materials tuned with negative SE domains can be explored to serve as superhydrophobic coatings capable of meeting engineering needs under harsh conditions in areas like aeronautics, astronautics, ships, and missiles.

## Supplementary Materials

Supplementary 1 Figure S1: transmission electron microscopy images and electron diffraction patterns of the powder stripped from the coating after the second stage of coating growth. Figure S2: contact angles dependence on pH and NaF concentration in the electrolyte. Figure S3: surface and cross-sectional morphologies of the superhydrophobic coating: A~C present irregular coral-like skeletons on the coating surface at different magnifications; D~F show cross-sectional images of the coating at different magnifications. Figure S4: (A1) XRD, (A2) SEM and EDS, and (B1-B4) XPS spectra of the superhydrophobic coating. Figure S5: hydrophobic characteristics of the disk made of powder scraped from the coating surface. Figure S6: the IR spectrums of samples after heating at different temperatures. Figure S7: bouncing test: (A) superhydrophobic coating and (B) hydrophilic coating. Figure S8: high-temperature test at 300°C: (A) heating apparatus; (B) superhydrophobic coating; (C) hydrophilic coating. Figure S9: burning test on polyimide (PI), polytetrafluoroethylene (PTFE), and the superhydrophobic coating. Figure S10: wear test. Figure S11: hydrophobic characteristics of the superhydrophobic coating after the salt spraying tests for 250 h and 370 h. The contact angles are still above 140 after 250 h and 370 h.

## References

[1] Lodziana Z., Topsoe N. Y., Norskov J. K. (2004). A negative surface energy for alumina. *Nature Materials*.

[2] Vishnoi M., Muthupandi V., Murugan S. S. (2017). Characterization of hydrophobic coating developed by micro arc oxidation on AA2014 alloy. *Indian Journal of Engineering and Materials Sciences*.

[3] Zhu X. F., Song Y., Liu L. (2009). Electronic currents and the formation of nanopores in porous anodic alumina. *Nanotechnology*.

[4] Yamaguchi G., Yasui I., Chiu W.-C. (1970). A new method of preparing *θ*-alumina and the interpretation of its X-ray-powder diffraction pattern and electron diffraction pattern. *Bulletin of the Chemical Society of Japan*.

[5] Li F., Zhang L., Metzger R. M. (1998). On the growth of highly ordered pores in anodized aluminum oxide. *Chemistry of Materials*.

[6] Ji S., Weng Y., Wu Z. (2017). Excellent corrosion resistance of P and Fe modified micro-arc oxidation coating on Al alloy. *Journal of Alloys and Compounds*.

[7] Mikeska K. R., Bennison S. J. (1999). Corrosion of alumina in aqueous hydrofluoric acid. *Journal of American Ceramic Society*.

[8] Cai S. H., Rashkeev S. N., Pantelides S. T., Sohlberg K. (2002). Atomic scale mechanism of the transformation of *γ*-Alumina to *θ*-Alumina. *Physical Review Letters*.

[9] Mchale J. M., Auroux A., Perrotta A. J., Navrotsky A. (1997). Surface energies and thermodynamic phase stability in nanocrystalline aluminas. *Science*.

[10] Liu T. Y., Kim C. J. (2014). Turning a surface superrepellent even to completely wetting liquids. *Science*.

[11] Vakarelski I. U., Patankar N. A., Marston J. O., Chan D. Y. C., Thoroddsen S. T. (2012). Stabilization of Leidenfrost vapour layer by textured superhydrophobic surfaces. *Nature*.

[12] Lu Y., Sathasivam S., Song J., Crick C. R., Carmalt C. J., Parkin I. P. (2015). Robust self-cleaning surfaces that function when exposed to either air or oil. *Science*.

[13] Lundy R., Byrne C., Bogan J. (2017). Exploring the role of adsorption and surface state on the hydrophobicity of rare earth oxides. *ACS Applied Materials & Interfaces*.

[14] Khan S., Azimi G., Yildiz B., Varanasi K. K. (2015). Role of surface oxygen-to-metal ratio on the wettability of rare-earth oxides. *Applied Physics Letters*.

[15] Hu D. L., Bush J. W. M. (2005). Meniscus-climbing insects. *Nature*.

[16] Wong T. S., Kang S. H., Tang S. K. Y. (2011). Bioinspired self-repairing slippery surfaces with pressure-stable omniphobicity. *Nature*.

[17] Erbil H. Y., Demirel A. L., Avci Y., Mert O. (2003). Transformation of a simple plastic into a superhydrophobic surface. *Science*.

[18] Genzer J., Efimenko K. (2000). Creating long-lived superhydrophobic polymer surfaces through mechanically assembled monolayers. *Science*.

[19] Zhai L., Cebeci F. C., Cohen R. E., Rubner M. F. (2004). Stable superhydrophobic coatings from polyelectrolyte multilayers. *Nano Letters*.

