# Supplementary material for: Tuning Superhydrophobic Materials with Negative Surface Energy Domains
Source: Research (Wash D C). 2019 Nov 30;2019:1391804. doi: 10.34133/2019/1391804 (PMC6944225; doi:10.34133/2019/1391804)
Supplement: Supplementary 1 — Figure S1: transmission electron microscopy images and electron diffraction patterns of the powder stripped from the coating after the second stage of coating growth. Figure S2: contact angles dependence on pH and NaF concentration in the electrolyte. Figure S3: surface and cross-sectional morphologies of the superhydrophobic coating: A~C present irregular coral-like skeletons on the coating surface at different magnifications; D~F show cross-sectional images of the coating at different magnifications. Figure S4: (A1) XRD, (A2) SEM and EDS, and (B1-B4) XPS spectra of the superhydrophobic coating. Figure S5: hydrophobic characteristics of the disk made of powder scraped from the coating surface. Figure S6: the IR spectrums of samples after heating at different temperatures. Figure S7: bouncing test: (A) superhydrophobic coating and (B) hydrophilic coating. Figure S8: high-temperature test at 300°C: (A) heating apparatus; (B) superhydrophobic coating; (C) hydrophilic coating. Figure S9: burning test on polyimide (PI), polytetrafluoroethylene (PTFE), and the superhydrophobic coating. Figure S10: wear test. Figure S11: hydrophobic characteristics of the superhydrophobic coating after the salt spraying tests for 250 h and 370 h. The contact angles are still above 140 after 250 h and 370 h. [file 1391804.f1.docx]

Tuning superhydrophobic materials with negative-surface-energy domains

Zhongzhen Wu^a^, Liangliang Liu^b^, Shunning Li^a^, Shunping Ji^a^, Pinghu Chen^a^, Suihan Cui^a^, Zhengyong Ma^a^, Yuchang Weng^a^, Qian Huang^a^, Zhongcan Wu^a^, Hao Wu^a^, Yuan Lin^a^, Ricky KY Fu^b^, Hai Lin^a^, Xiubo Tian^a^, Paul K Chu^b^ and Feng Pan^a, ^[[1]](#footnote-1)^*^

^a^ School of Advanced Materials, Peking University Shenzhen Graduate School, Shenzhen 518055, China

^b^ Department of Physics and Materials Science, City University of Hong Kong, Tat Chee Avenue, Kowloon, Hong Kong, China

**Support information**


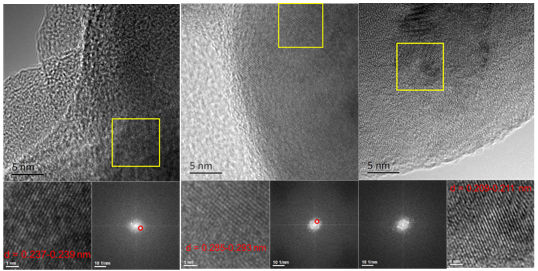


Fig. S1 Transmission electron microscopy images and electron diffraction patterns of the powder stripped from the coating after the second stage of coating growth.


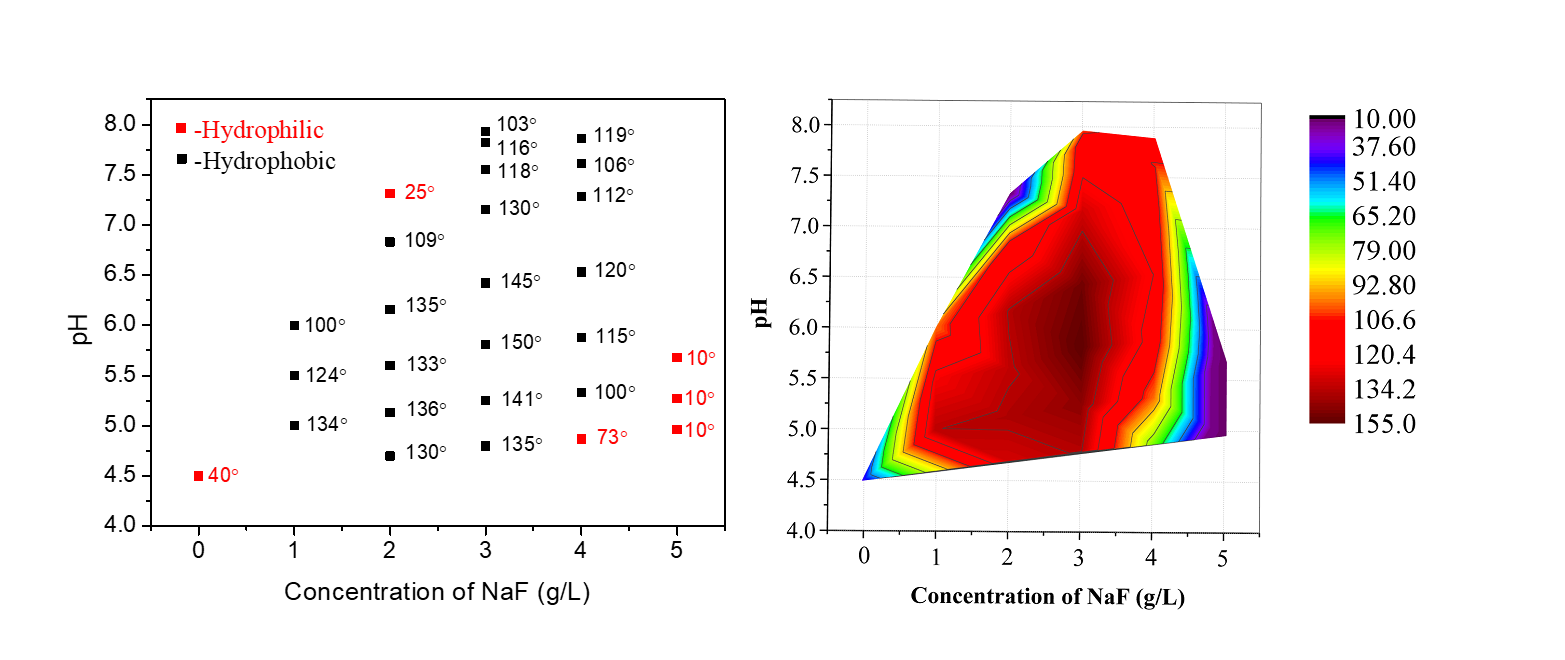


Fig. S2 Contact angles dependence on pH and NaF concentration in the electrolyte.


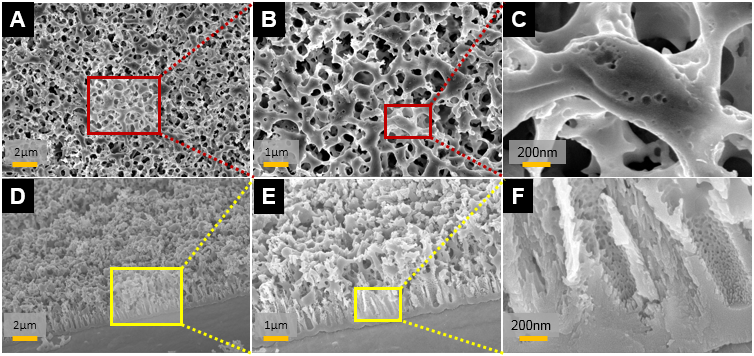


Fig. S3 Surface and cross-sectional morphologies of the superhydrophobic coating: A~C present irregular coral-like skeletons on the coating surface at different magnification; D~F show cross-sectional images of the coating at different magnification.


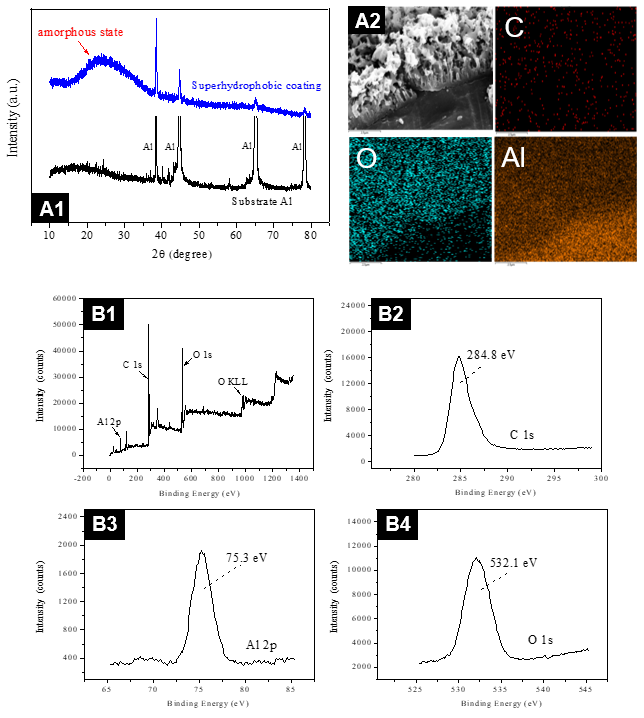


Fig. S4 (A1) XRD, (A2) SEM and EDS, and (B1-B4) XPS spectra of the superhydrophobic coating.


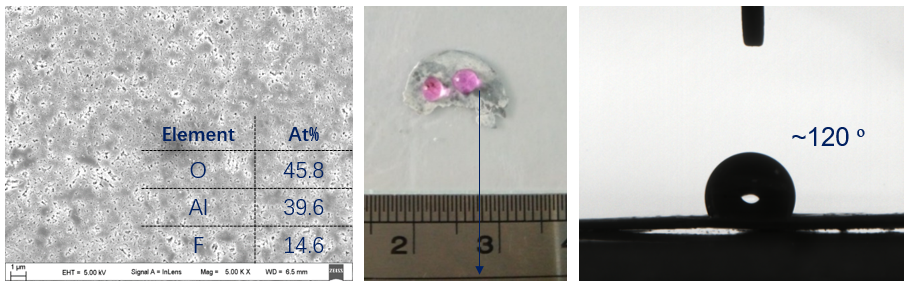


Fig. S5 Hydrophobic characteristics of the disk made of powder scraped from the coating surface.


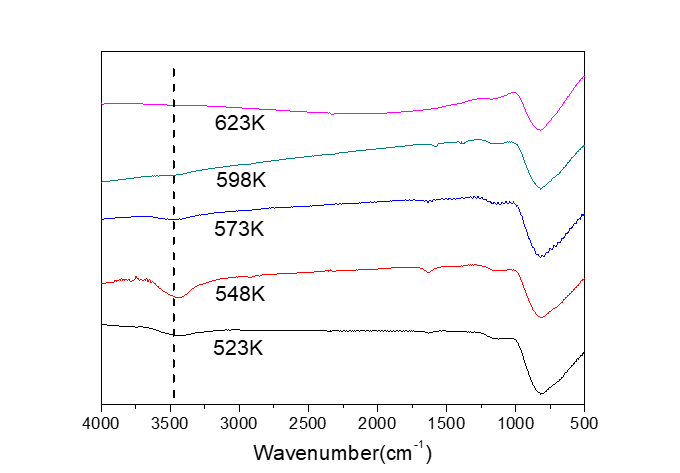


Fig.S6 The IR spectrums of samples after heating at different temperatures.


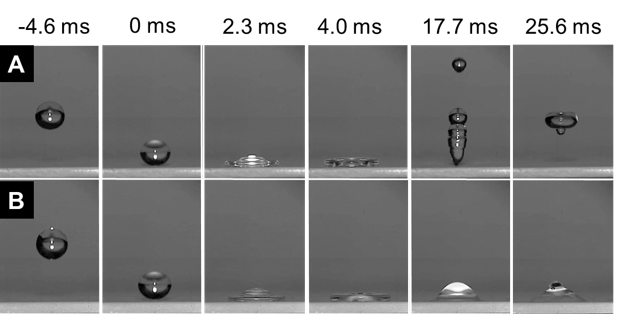


Fig. S7 Bouncing test: (A) Superhydrophobic coating and (B) Hydrophilic coating.


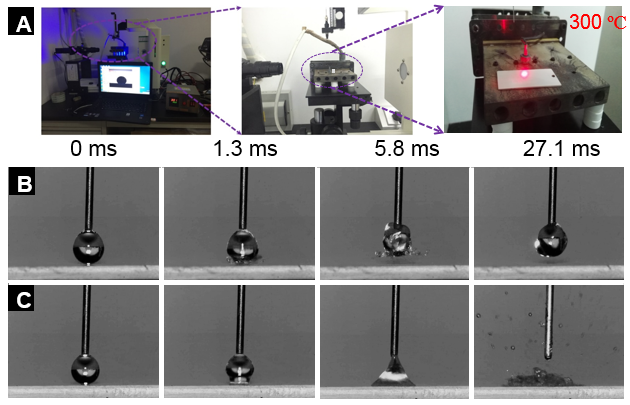


Fig. S8 High-temperature test at 300℃: (A) [H](javascript:;)eating [apparatus](javascript:;); (B) Superhydrophobic coating; (C) Hydrophilic coating.


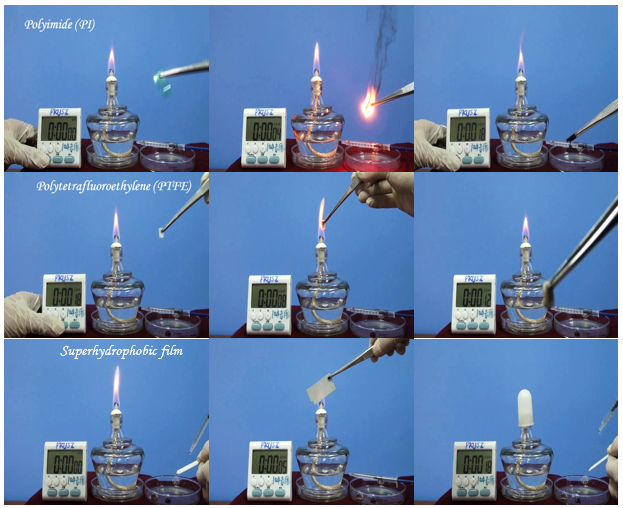


Fig. S9 Burning test on polyimide (PI), polytetrafluoroethylene (PTFE), and the superhydrophobic coating.


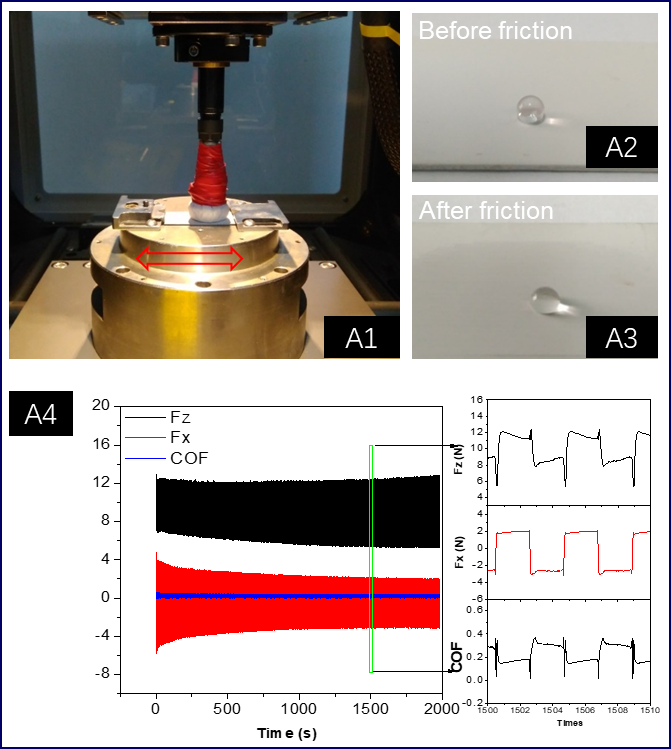


Fig. S10 Wear test.


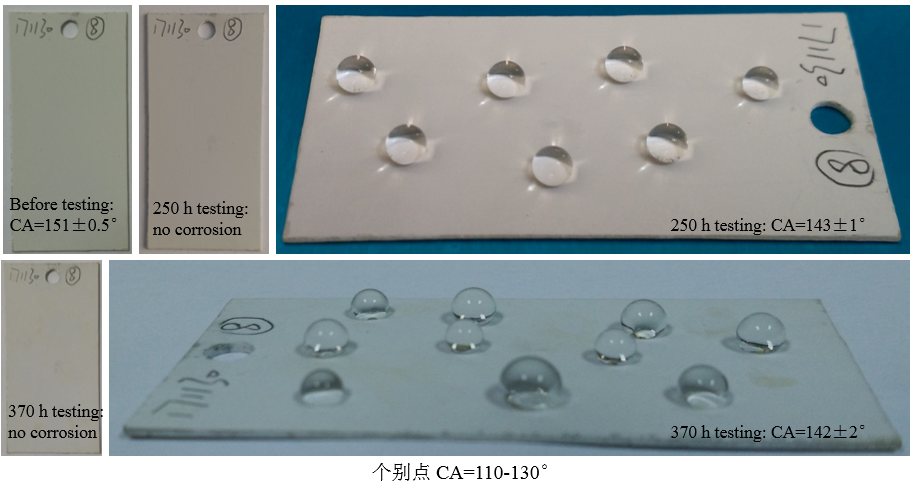


Fig. S11 Hydrophobic characteristics of the superhydrophobic coating after the salt-spraying tests for 250 h and 370 h. The contact angles are still above 140 after 250 h and 370 h.

1. * Corresponding authors. Tel/Fax: +86-755-26032957; *E-mail address:* Panfeng@pkusz.edu.cn (F. Pan) [↑](#footnote-ref-1)
